# Supposed Death

**Published:** 1846-04

**Authors:** 


11. *Supposed Death.*—Mr. A. S. Taylor mentions the following extraordinary case:—In October, 1830, a servant girl, who had retired to bed in apparently perfect health, was found the following morning, as it was supposed, dead. A surgeon who was sent for pronounced her to be certainly dead, and that she had probably been so some hours. A coroner's inquest was summoned for four o'clock of the same day to inquire into the cause of death, and directions were given that a post-mortem inspection of the body should be made in the meantime. The reporter of the case was requested to give his assistance. Accompanied by the surgeon who had been consulted, he went to the house about two o'clock for the purpose of making the inspection. The deceased was found lying on the bed, in an easy posture, on her left side, her body forming somewhat of a semi-circle. The countenance was pallid, but so perfectly placid and composed as to give her the appearance of being in a deep sleep. The heat of the body, although she must have been dead eight or ten hours, was not in the least diminished. The room was carefully searched, but nothing in the shape of poison, nor any other means of self-destruction, could be discovered. Every article of apparel lay around as it might be supposed to have been left by a person going to bed in perfect health as usual. The heat of the body not diminishing a vein was opened, and various stimuli applied, but without producing any sign of resuscitation. The respiratory and circulatory processes had ceased; no artery could be felt pulsating. Two hours had now elapsed since their arrival, and the parties still hesitated to perform the inspection, when a message was sent to them stating that the jury were waiting for their evidence. The inspection was then commenced, but on moving the body for the purpose, the warmth and pliancy of the limbs were such, as to give the examiners the idea that they were inspecting a living subject. The internal cavities were so warm that a very copious steam issued from them when they were laid open. All the viscera were healthy, there were no signs of disease; nothing appeared to account for death, and from what they saw the inspectors regretted that they had not postponed the examination until the signs of death had been completely manifested. For obvious reasons, the name of the place where this extraordinary case occurred, and the name of the reporter, were suppressed. He had evidently communicated the details in a fit of remorse for his precipitancy.—*Lond. Med. Gaz.* in *Med. News and Library.*

